# Nr4a1 promotes renal interstitial fibrosis by regulating the p38 MAPK phosphorylation

**DOI:** 10.1186/s10020-023-00657-y

**Published:** 2023-05-09

**Authors:** Yilin Tao, Chengyuan Tang, Ju Wei, Yi Shan, Xi Fang, Ying Li

**Affiliations:** 1 Department of Nephrology, The Second Xiangya Hospital, Central South University, Changsha, 410011 Hunan China; 2 Key Laboratory of Kidney Disease and Blood Purification in Hunan Province, Changsha, 410011 Hunan China

**Keywords:** Renal tubulointerstitial fibrosis, Nuclear receptor subfamily 4 group a member 1, p38 MAPK, Cytosporone B

## Abstract

**Background:**

Renal interstitial fibrosis (RIF) is a common pathway to end-stage renal disease regardless of the initial etiology. Currently, the molecular mechanisms for RIF remains not fully elucidated. Nuclear receptor subfamily 4 group A member 1(Nr4a1), a member of the NR4A subfamily of nuclear receptors, is a ligand-activated transcription factor. The role of Nr4a1 in RIF remains largely unknown.

**Methods:**

In this study, we determined the role and action mechanism of Nr4a1 in RIF. We used unilateral ureteral obstruction (UUO) mice and transforming growth factor (TGF)-β1-treated human renal proximal tubular epithelial cells (HK-2 cells) as in vivo and in vitro models of RIF. A specific Nr4a1 agonist Cytosporone B (Csn-B) was applied to activate Nr4a1 both in *vivo* and in *vitro*, and Nr4a1 small interfering RNA was applied in vitro. Renal pathological changes were evaluated by hematoxylin and eosin and Masson staining, and the expression of fibrotic proteins including fibronectin (Fn) and collagen-I (Col-I), and phosphorylated p38 MAPK was measure by immunohistochemical staining and western blot analysis.

**Results:**

The results showed that Nr4a1 was upregulated in UUO mouse kidneys, and was positively correlated with the degree of interstitial kidney injury and the levels of fibrotic proteins. Csn-B treatment aggravated UUO-induced renal interstitial fibrosis, and induced p38 MAPK phosphorylation. In *vitro*, TGF-β induced Nr4a1 expression, and Nr4a1 downregulation prevented TGF-β1-induced expression of Fn and Col-I and the activation of p38 MAPK. Csn-B induced fibrotic proteins expression and p38 MAPK phosphorylation, and moreover Csn-B induced fibrotic proteins expression was abrogated by treatment with p38 MAPK inhibitor SB203580. We provided further evidence that Csn-B treatment promoted cytoplasmic accumulation of Nr4a1.

**Conclusion:**

The findings in the present study indicate that Nr4a1 promotes renal fibrosis potentially through activating p38 MAPK kinase.

**Supplementary Information:**

The online version contains supplementary material available at 10.1186/s10020-023-00657-y.

## Introduction

Renal interstitial fibrosis (RIF), the common pathway for the progression of all chronic kidney diseases (CKD) to end stage renal failure. The pathogenesis of RIF is complex and involves a variety of cell types, including activation of interstitial fibroblasts and pericytes, phenotypic transformation of tubule epithelial cells and endothelial cells, as well as renal infiltration of circulating myofibroblasts and inflammatory cells(Liu 2011). Previous studies have shown that under the condition of profibrotic stimulation, renal tubule epithelial cells undergo a variety of reactions including dedifferentiation, cell-cycle arrest, autophagy, apoptosis and metabolic changes, and can also act like inflammatory cells and fibroblasts that produce bioactive molecules, eventually driving interstitial inflammation and fibrosis(Liu et al. 2018; Gewin 2018; Li et al. 2019; Docherty et al. 2006). As such, prevention or treatment of renal tubular injury is critical to impede the development of kidney disease. The search for novel therapeutic targets in renal tubular epithelial cells for interstitial fibrosis is of great importance to prevent the development of chronic kidney disease.

Recent studies indicated an association between TGF-β1 and nuclear receptor subfamily 4 group A member 1(Nr4a1) (Palumbo-Zerr et al. 2015; Hedrick and Safe 2017; Hedrick et al. 2018; Shrestha et al. 2020). Nr4a1 is a transcription factor which belongs to the nuclear hormone receptor (NR) subfamily. As an early response gene, Nr4a1 is rapidly induced by a diverse range of stimulators including growth factors, cytokines, neurotransmitters and stress(Maxwell and Muscat 2006), and has been implicated in various biological events such as apoptosis, proliferation, inflammation and metabolism(Wu et al. 2019; Estrada et al. 2020; Xiong et al. 2020). In 2015, Palumbo-Zerr et al. (2015). However, Zhong et al. demonstrated that Nr4a1 promoted TGF-β-induced breast cancer cell epithelial-to-mesenchymal transition (EMT) and cell migration both in *vitro* and in *vivo*(Hedrick and Safe 2017). Nr4a1 was also shown to facilitate TGF-β-induced lung cancer cells invasion and embryonal rhabdomyosarcoma cells invasion(Hedrick et al. 2018; Shrestha et al. 2020). In the kidney, suppression of Nr4a1 expression was shown to ameliorate renal ischemia-reperfusion injury (IRI)(Shi et al. 2019). In diabetic nephropathy, Nr4a1 levels were positively related to renal fibrosis and glomerular apoptosis, and its induction contributed to high glucose-induced mitochondrial damage in human renal mesangial cells(Sheng et al. 2018). However, the role and action mechanism of by Nr4a1 in renal interstitial fibrosis remain largely unclear.

Activation of mitogen-activated protein kinases (MAPKs) has been implicated in renal fibrogenesis(Rhyu et al. 2005). p38 MAPK, a subclasses of MAPKs, is a stress-activated protein kinase. In response to cellular stresses, p38 MAPK kinase can translocate to the nucleus and then induce the expression of downstream target genes. It has been reported that activation of MAPK signaling pathway promoted epithelial-to-mesenchymal transition (EMT) in a unilateral ureteral obstruction (UUO) rat model of renal fibrosis and in TGF-β1-treated renal tubular epithelial cells, and inhibition of MAPK signaling prevented EMT and delayed renal fibrosis(Rhyu et al. 2005; Hung et al. 2016; Ma et al. 2009). We hypothesized that Nr4a1 may activate MAPK signaling pathway and therefore promote renal fibrosis. To verify our hypothesis, in the present study, we determined the role of Nr4a1 in renal fibrosis and the involvement of p38 MAPK in Nr4a1-mediated functions by using UUO mice and TGF-β1-treated HK-2 cells as in vivo and in vitro models of renal fibrosis. We showed that Nr4a1 was upregulated in UUO mouse kidneys, and was positively correlated with the degree of renal fibrosis. Nr4a1 downregulation prevented TGF-β1-induced fibrotic changes and activation of p38 MAPK in renal tubular cells. The specific Nr4a1 agonist Cytosporone B (Csn-B) promoted renal fibrosis, and induced p38 MAPK phosphorylation. Csn-B induced fibrotic proteins expression in renal tubular cells was abrogated by treatment with p38 MAPK inhibitor SB203580. Csn-B treatment promoted cytoplasmic accumulation of Nr4a1. The findings in the present study indicate that Nr4a1 promotes renal fibrosis potentially through activating p38 MAPK kinase.

## Materials and methods

### Animal models and treatment

Animal experiments were approved by the Institutional Animal Care and Use Committee (IACUC) at the Second Xiangya Hospital of Central South University (No.2022 0509). Male C57BL/6 mice (8 weeks old) were purchased from Slyke Jingda (Hunan, China). Mice were housed in a pathogen-free condition under cycles of 12:12-h light and dark with free access to food and water. To perform unilateral ureteral obstruction (UUO) surgery, mice were anesthetized by intraperitoneal injection of pentobarbital at 60 mg/kg of body weight and then fixed on a clean operating table. The abdomen was opened in the middle, the left kidney was exposed, and then the left ureter was separated. Ligation was conduced respectively at both proximal and distal ureters, and the ureter was cut between the two ligation sites. For sham operation, the ureters were separated without ligation. Csn-B was dissolved in DMSO to make a stock solution of 120 mg/ml, and the stock solution was diluted with corn oil to reach a working concentration of 2 mg/ml. For treatment, 50 mg/kg of Csn-B or vehicle (DMSO + corn oil) were administered via oral gavage starting 7 days before UUO surgery to 7 days after surgery, once every other day. The mice were sacrificed 7 days after the operation, and the renal tissues were collected for the subsequent histological and biochemical analysis.

### Renal pathological analysis

Renal pathology was evaluated via hematoxylin and eosin (H&E), Masson and PAS staining. Mouse kidney tissue was embedded in paraffin. For H&E staining, after deparaffinization and dehydration, cell nuclear and cytoplasm of kidney sections were respectively labeled by hematoxylin and eosin according to manufacture`s introduction (Servicebio, China). For Masson staining, renal sections were stained with Lixin red, phosphomolybdic acid treatment, and aniline blue according to manufacture`s introduction (Servicebio, China). For PAS staining, the sections were oxidized with periodic acid and then stained with Schiff reagent (Servicebio, China).

### Immunohistochemical (IHC) analysis

For immunohistochemical analysis, the paraffin-embedded renal sections undergone deparaffinization and dehydration. The kidney sections were placed in sodium citrate buffer and then boiled for 8 min at high heat and 20 min at medium heat in a microwave oven for antigen retrieval. After cooling down to room temperature, the slices were sequentially incubated with 3% H2O2, goat serum, and primary antibody against Nr4a1(proteintech, 25851-1-AP)、Fibronectin(Fn)(Abcam, ab2413)、 Collagen I (Col-I)(Affinity, AF7001)、F4/80(proteintech, 27044-1-AP)、α-SMA(proteintech, 14395-1-AP) at 4℃ overnight. After incubation with a corresponding horseradish peroxidase (HRP)-conjugated secondary antibody at room temperature for 1 h, the slices were stained with DAB chromogenic solution, and the images were observed under phase contrast microscope.

### Semi-quantitative analysis of Masson staining and IHC analysis

The results of Masson staining and IHC analysis were quantified via evaluating the percentage of positive area in a blinder manner (the person who collected images was different from the person who performed quantitate analysis, and both of them were blinded to the experimental groups). For quantification, we randomly selected 10 fields of view (200x) of the kidney tissue section of each mouse. The ratio of the positive area was analyzed using Image J software.

### Cell culture, transfection and treatment

HK-2 cells were obtained from the Nephrology Institute of the Second Xiangya Hospital of Central South University. The cells were cultured in an incubator (37℃, 5%CO2) and with DMEM (Dulbecco’s Modified Eagles’s Medium) containing 10% FBS (fetal bovine serum). For transfection, the cells were planted in 35 mm culture dishes to reach a confluence of around 40%, and transfection of short small RNA (siRNA) was performed with lip2000 according to the manufacture`s instruction(Invitrogen, United States). For chemical treatments, the cells were planted in 35 mm cell culture dishes to reach around 50% cell confluence, and the cells were then treated with 5ng/ml TGF-β1, 10ug/ml Csn-B or vehicle (DMSO) for another 24 h. For treatment with p38 MAPK inhibitor SB203580, the cells were pretreated with 10 μm SB203580 for 0.5 h, and were then cotreated with Csn-B for another 24 h.

### Western blot analysis

Cells were washed with PBS and lysed with RIPA lysis buffer containing protease inhibitor and phosphatase inhibitor. Protein concentration was measured by the BCA method with reagents from Thermo Scientific. Equal amounts of proteins were separated by electrophoresis using 8-10% polyacrylamide gel and were then transferred to PVDF membrane. After incubation with 5% skim milk, the membranes were sequentaly incubated with a primary antibody at 4℃ overnight and a corresponding HRP-linked secondary antibody. The target protein was visualized using chemiluminescent substrate (Thermo Scientific). The protein band intensity was quantified by the ImageJ software (NIH).

### Immunofluorescence staining

Cells were washed with phosphate-buffered saline (PBS) and then fixed with 4% paraformaldehyde at room temperature for 15 min. The cells were permeabilized and blocked with 5% BAS in 0.1% Triton X-100 for 1 h. After incubation with a primary antibody overnight at 4℃, the cells were washed with PBS and then incubated with a corresponding Alexa-conjugated secondary antibody. Images were obtained with a fluorescence microscope (Leika).

### Cytoplasmic protein extraction

Cytoplasmic protein extraction was performed with the Nuclear and Cytoplasmic Protein Extraction Kit from Beyotime (China) according to the manufacturer’s instruction. Briefly, the cells were scraped and then collected in PBS. After centrifugation, the precipitated cells were incubated with cytoplasmic protein extraction reagent containing PMSF and were then completely dispersed via vortex. The cell lysates were centrifuged at 4℃, 14,000 rcf for 20 min, and the cytoplasmic proteins were in supernatant.

### Statistical analysis

All data were expressed as mean ± standard deviation (SD), and statistical analysis was performed by GraphPad Prism software. Statistical differences between 2 groups were determined by the unpaired t test. And one-way analysis of variance (ANOVA), followed by Tukey’s post-tests, was used to determine the statistical differences among groups. Pearson’s correlation analysis was used to assess the correlation between two variables. Values of P<0.05 between groups indicated that the difference was statistically significant.

## Results

### Nr4a1 is upregulated in UUO kidneys

**Fig. 1:**
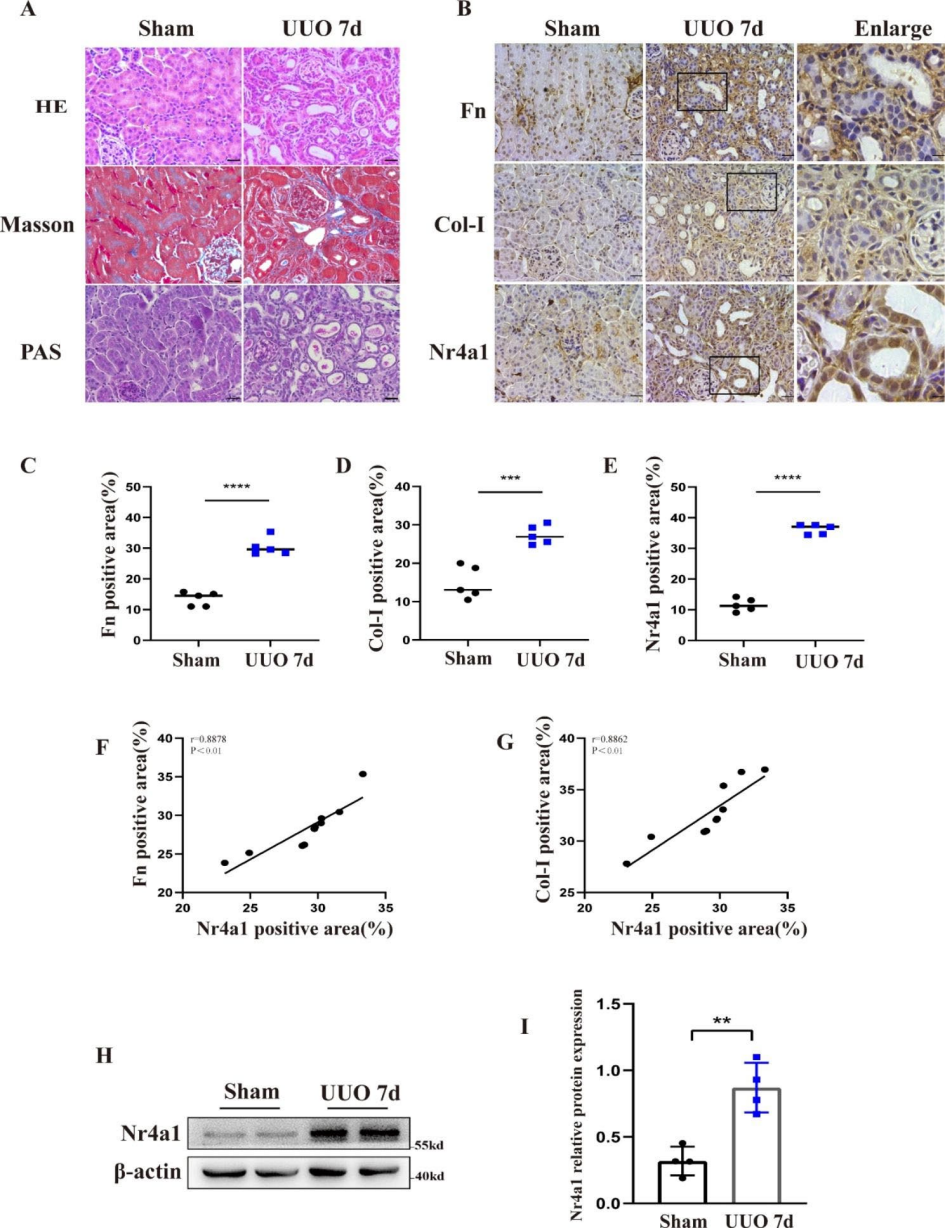
**Nr4a1 is elevated in the kidney of UUO mice** (**A**) Renal pathological analysis by H&E staining, Masson staining and PAS staining (400x, Bar: 50 μm). (**B**) Immunohistochemical staining of the expression of fibrotic proteins Fn, Col-I and Nr4a1 and (**C-E**) semi-quantitative analysis of positive areas. (**F-G**) Correlation analysis of Nr4a1expression with Fn and Col-I expression. (**H**) Western blot analysis of Nr4a1 expression in kidney tissues and (**I**) corresponding quantitative analysis. Unpaired t test was used to compare the statistical differences among sham and UUO 7d groups. *p<0.05, **p<0.01, ***p<0.001, ****p<0.0001

We then examined the expression of Nr4a1 in the kidney of a mouse model of UUO-induced renal fibrosis. 7 days after UUO or sham operation, the mice were sacrificed for collection of kidney tissues. Pathological analysis by H&E staining, Masson staining and PAS staining demonstrated that compared with the sham-operated mice, the UUO mice showed renal tubular dilatation, renal tubular atrophy, and renal infiltration of inflammatory cells and collagen deposition (Fig. 1A). Immunohistochemical staining showed that UUO mice had a dramatic increase of the levels of profibrotic proteins including Fn and Col-I in the kidneys compared with control mice (Fig. 1B and E). UUO mice also had a marked increase of Nr4a1 levels, and the Nr4a1 induction mainly happened in dilated renal tubules. A linear static analysis indicated that the levels of Nr4a1 was positively correlated with the levels of both Fn and Col-I (Fig. 1F-G). Western blot analysis confirmed the induction of Nr4a1 in obstructed kidney tissues (Fig. 1H-I).

### Treatment with Nr4a1 agonist Csn-B exacerbates renal interstitial fibrosis in UUO kidneys

**Fig. 2:**
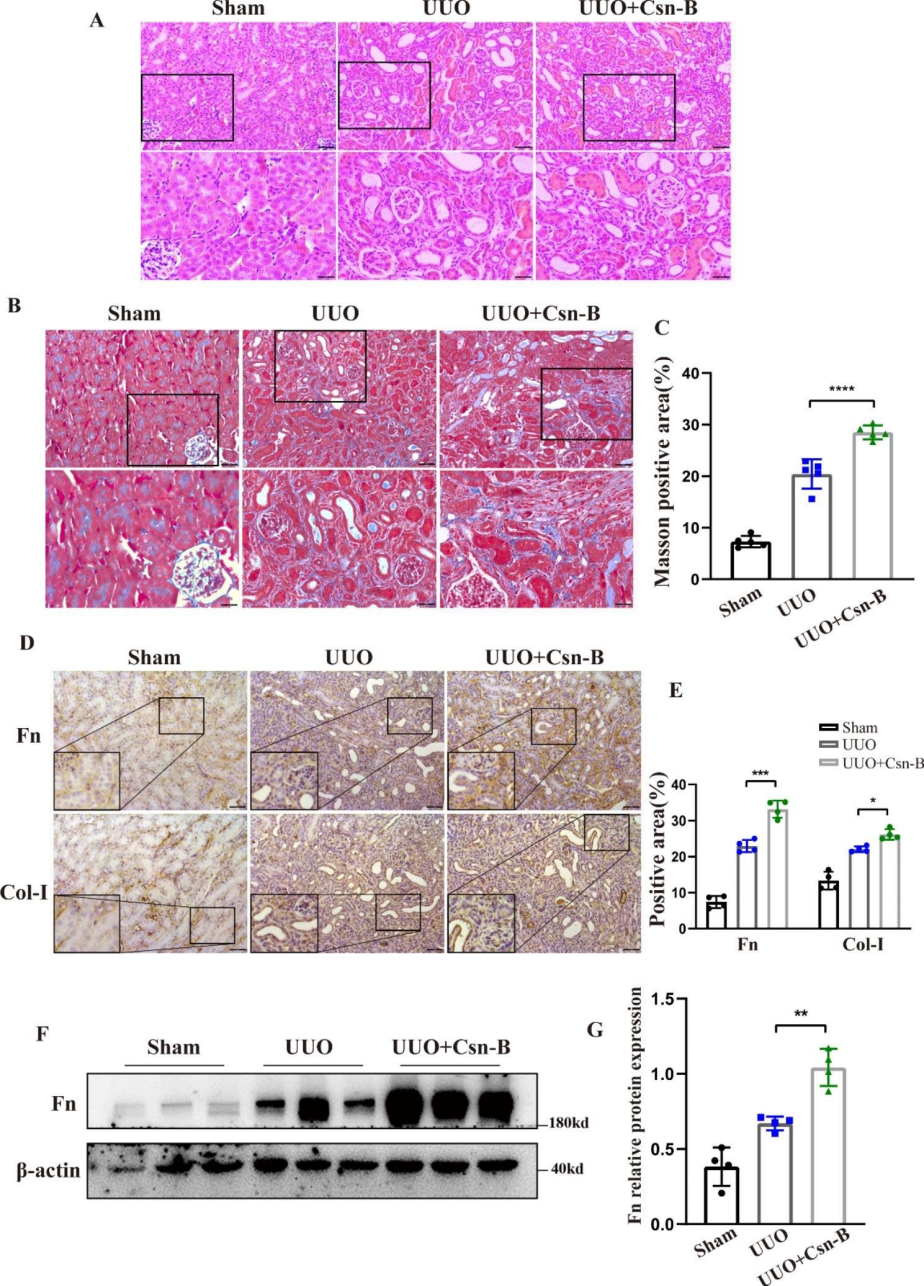
**Csn-B aggravates renal interstitial fibrosis in UUO mice** (**A**) H&E staining of kidney tissues (200x/400x, Bar: 50 μm) (**B**) Masson staining detecting collagen deposition in kidney tissues (200x/400x, Bar: 50 μm). (**C**) Quantitative analysis of the results of Masson staining. (**D**) Immunohistochemical analysis of Fn and Col-I in kidney tissue(200x/400x, Bar: 50 μm). (**E**) Quantitative analysis of the expression of Fn and Col-I. (**F**) Immunoblot blot of Fn. (**G**) Quantitative analysis of the expression of Fn expression. One-way analysis of variance (ANOVA), followed by Tukey’s post-tests, was used to compare the statistical differences among sham、UUO and UUO + Csn-B groups.*p<0.05, **p<0.01, ***p<0.001

To investigate the role of Nr4a1 in renal interstitial fibrosis in vivo, we evaluated the effect of a specific Nr4a1 agonist Csn-B on the renal pathologies in UUO mice. H&E staining showed that Csn-B treatment aggravated UUO-induced tubular injury (Fig. 2A). Masson staining and quantitative analysis showed that Csn-B treatment increased collagen deposition in UUO kidneys (Fig. 2B-C). Immunohistochemical analysis also showed that UUO mice treated with Csn-B had significantly higher levels of Fn and Col-I in the kidneys compared to UUO mice without Csn-B treatment (Fig. 2D-E). Western blot analysis confirmed the Csn-B-increased expression of Fn in UUO kidneys (Fig. 2F-G). Immunohistochemical analysis showed that in UUO mice, Csn-B treatment enhanced renal infiltration of macrophage. Western blot analysis also showed that Csn-B treatment enhanced the expression of α-SMA in UUO mice (Supplement Fig. 1), and expression of neutrophil gelatinase-associated lipocalin (NGAL), a marker of renal tubular damage in the kidneys (Supplement Fig. 2). Collectively, these findings indicate that Csn-B aggravates UUO-associated renal interstitial fibrosis.

**Fig. 3:**
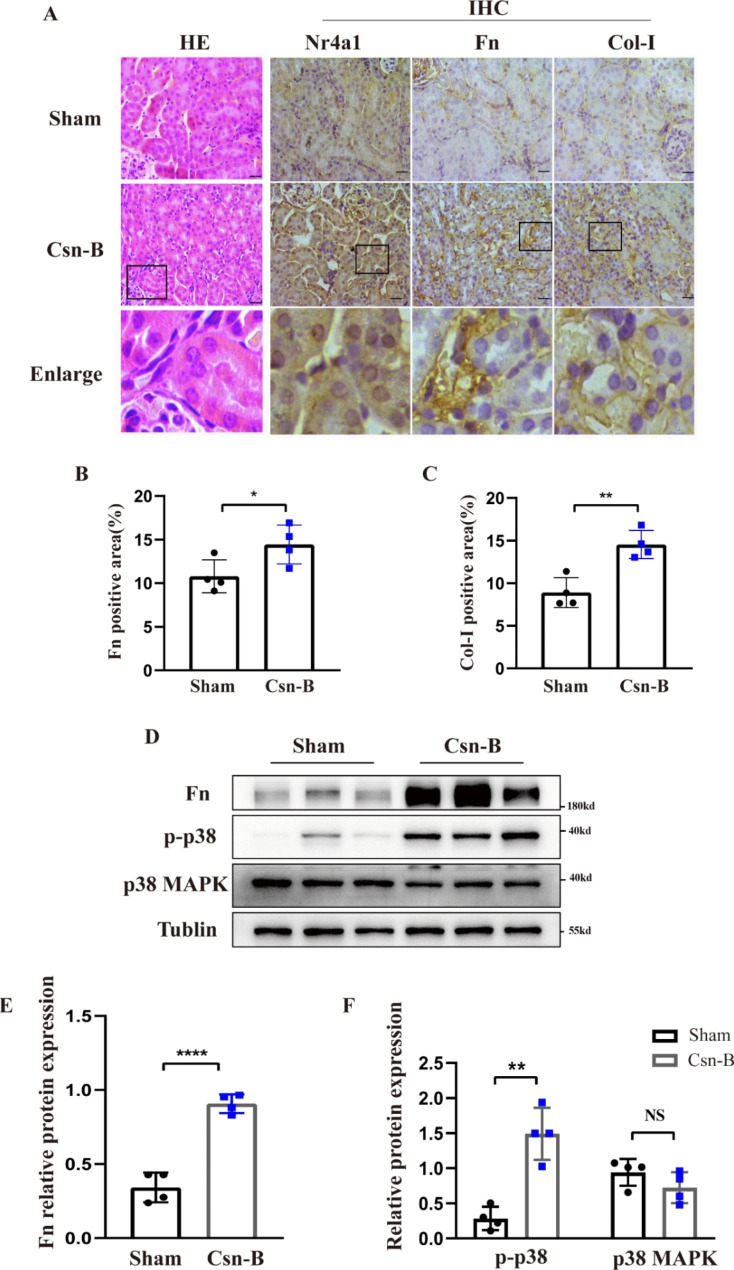
**Csn-B induces fibrotic protein expression and p38 MAPK phosphorylation** (**A**) H&E staining detecting pathological changes and immunohistochemical analysis of the expression of Nr4a1 and fibrotic proteins Fn, Col-I. (**B-C**) Semi-quantitative analysis of positive areas of FN or Col-I staining. (400x, Bar: 50 μm) (**D**) Western blot analysis of and (**E-F**) corresponding quantitative analysis of the expression of Fn and phosphorylated p38 MAPK in renal tissues. Unpaired t test was used to compare the statistical differences between sham and Csn-B groups. *p<0.05, **p<0.01

We future determined whether Csn-B treatment alone induced renal fibrosis in mice. H&E staining showed that compared to vehicle treatment, Csn-B treatment caused no apparent renal pathologies like dilated or atrophic tubular epithelial cells. Immunohistochemical analysis demonstrated that Csn-B treatment alone increased the expression of Nr4a1 and fibrotic protein Fn and Col-I in the kidneys of mice (Fig. 3A-C). In addition, we found that Csn-B treatment alone induced renal infiltration of macrophage, renal expression of myofibroblasts α-SMA (Supplement Fig. 3) and NGAL (Supplement Fig. 4). Western blot analysis indicated that Csn-B treatment alone also caused an increase in the levels of Fn and phosphorylated p38 MAPK (Fig. 3D-F). Collectively, these results suggest that Csn-B treatment alone also increase the expression of fibrotic proteins and p38 MAPK activation.

### Nr4a1 promotes TGF-β1-induced fibrotic effect in HK-2 cells

**Fig. 4:**
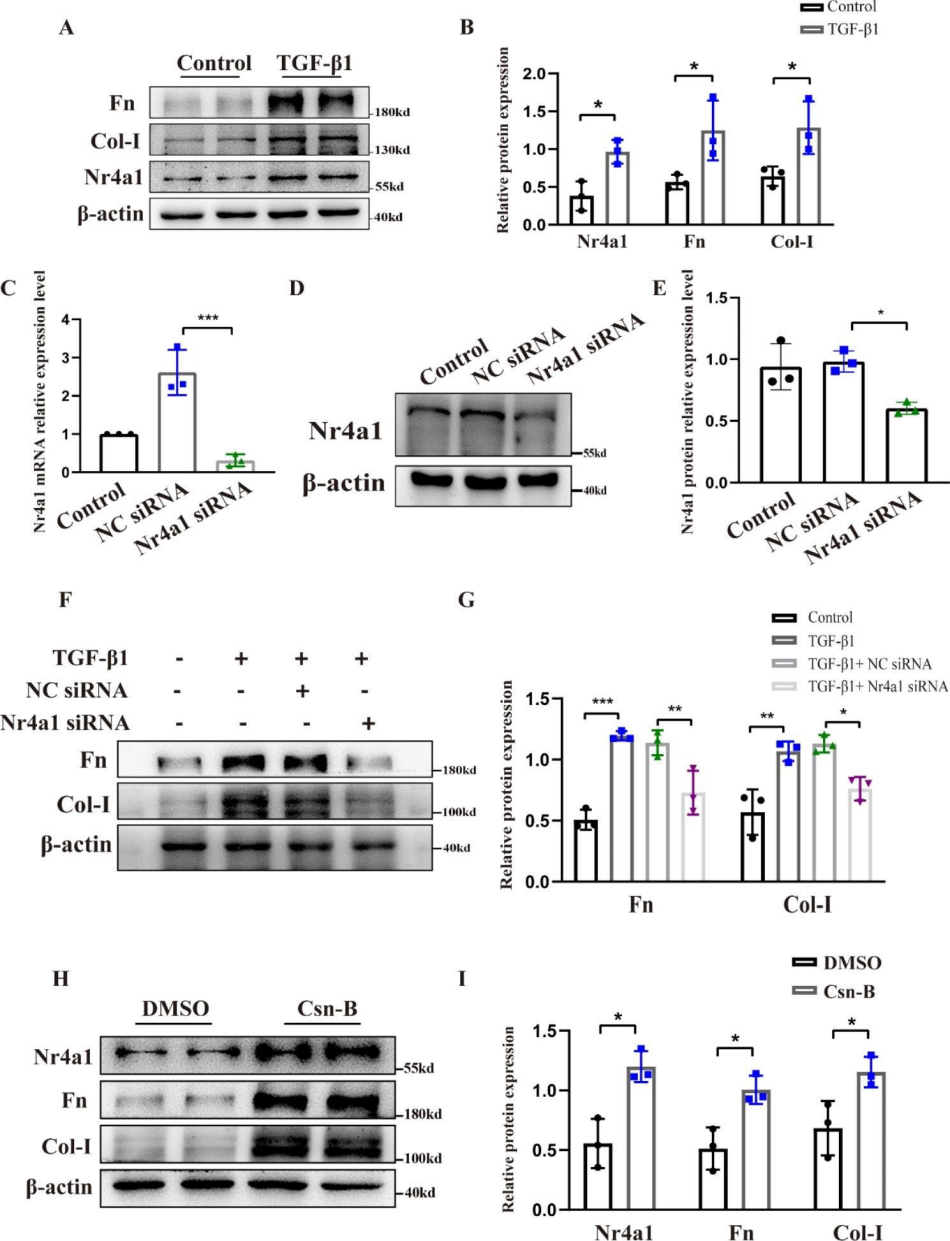
**Nr4a1 promotes TGF-β1-induced fibrotic effect in HK-2 cells** (**A**) Western blot analysis of Nr4a1, Fn, Col-I expression in HK-2 cells with or without TGF-β1. (**B**) Quantitative analysis of the expression of targeted proteins in (A). (**C**) qRT-PCR analysis of the expression of Nr4a1 mRNA in HK-2 cells transfected with Nr4a1 siRNA or NC siRNA. (**D**) Western blot analysis of the expression of Nr4a1 mRNA in HK-2 cells transfected with Nr4a1 siRNA or NC siRNA. (E) Quantitative analysis of the expression of targeted proteins in (D). (**E**) Western blot analysis and (**F**) corresponding quantitative analysis of Fn and Col-I expression in HK-2 cells transfected with Nr4a1 siRNA after TGF-β1 treatment. (**G**) Western blot analysis and (**H**) corresponding quantitative analysis of Nr4a1, Fn, Col-I expression in Hk-2 cells with or without 10ug/ml Csn-B treatment. Statistical differences between two groups were determined by the unpaired t test. And one-way analysis of variance (ANOVA), followed by Tukey’s post-tests, was used to determine the statistical differences among groups. *p<0.05, **p<0.01, ***p<0.001

We also determined the role of Nr4a1 in a cell model of renal fibrosis that is induced by TGF-β1 treatment. To this end, the human proximal tubular cell line HK-2 cells were treated with 5ng/ml TGF-β1 as previously described (Hou et al. 2015). Western blot analysis showed that TGF-β1 treatment caused a dramatic increase of Nr4a1 and fibrotic proteins Fn and Col-I (Fig. 4A-B). Suppression of Nr4a1 expression with specific siRNA (Fig. 4C-E) inhibited TGF-β1-induced production of Fn and Col-I (Fig. 4F-G). The effect of Csn-B treatment on the expression of Nr4a1 and fibrotic proteins in HK-2 cells was also evaluated. Initially, we treated HK-2 cells with different concentration of Csn-B (1, 5, and 10 ug/ml) for 24 h, and found that 10 ug/ml of Csn-B caused a significant increase of Nr4a1 accompanied with increases of Fn and Col-I (Supplement Figs. 4H-I and 5A-D). 20 ug/ml and 30 ug/ml of Csn-B caused cell loss (Supplement Fig. 5E-F), and thus we choose the concentration of 10 ug/ml in following studies. Suppression of Nr4a1 expression by siRNA significantly suppressed Csn-B-induced expression of Fn and Col-I (Supplement Fig. 5C-D). Collectively, these findings indicate that activation of Nr4a1 promotes the fibrotic change of renal tubular cells in renal fibrosis.

### Inhibition of p38 MAPK kinase prevents the profibrotic effect of Nr4a1 in HK-2 cells

**Fig. 5:**
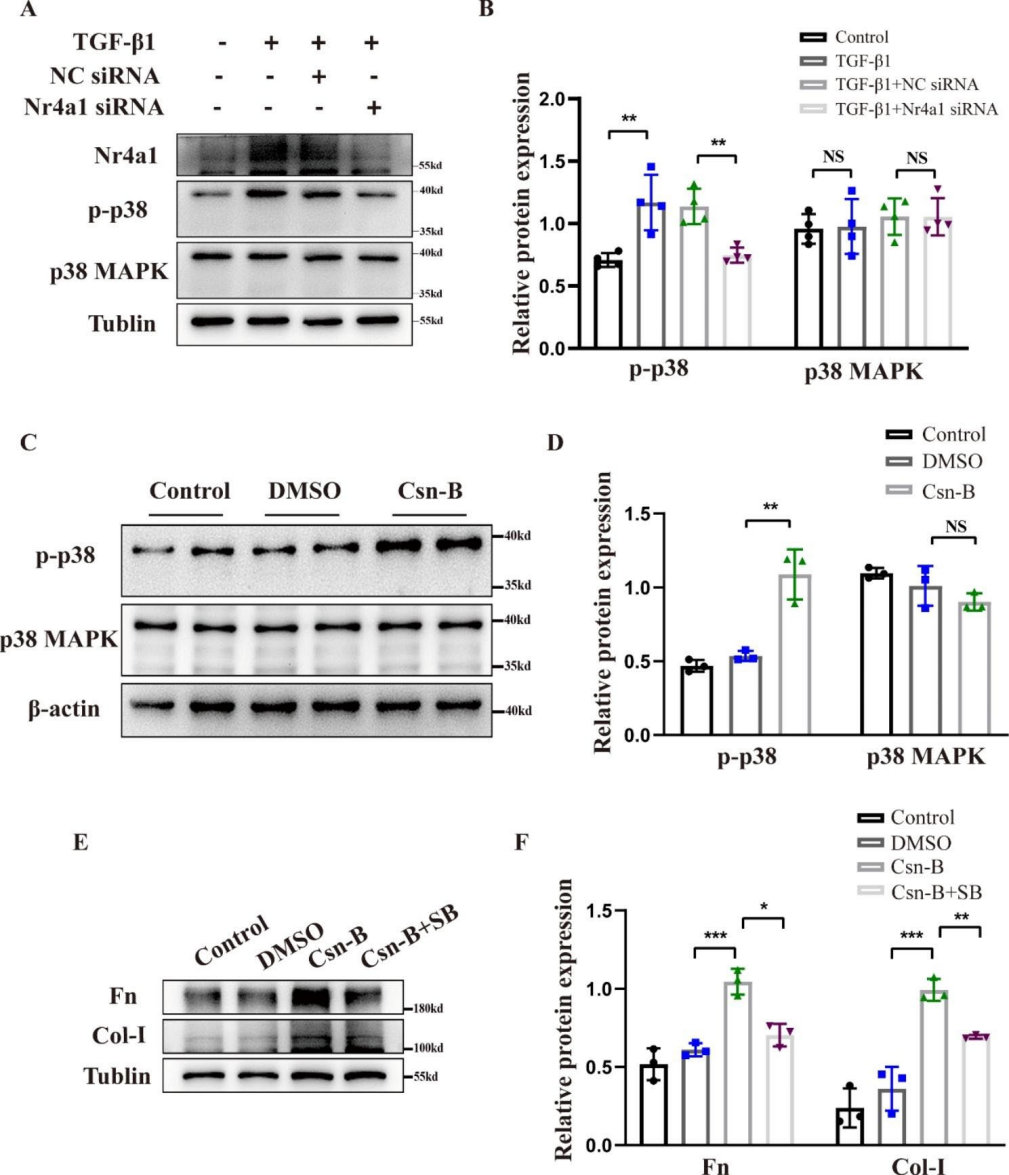
**Inhibition of p38 MAPK kinase prevents the profibrotic effect of Nr4a1 in HK-2 cells** (**A**) Western blot analysis and (**B**) corresponding quantitative analysis of p38 MAPK phosphorylation in HK-2 cells with or without Nr4a1 siRNA expression and TGF-β1 treatment. (**C**) Western blot analysis and (**D**) corresponding quantitative analysis of p38 phosphorylation level in HK-2 cells with or without Csn-B treatment. (**E**) Western blot analysis and (**F**) corresponding quantitative analysis of Fn and Col-I in HK-2 treated with Csn-B and p38 inhibitor. One-way analysis of variance (ANOVA), followed by Tukey’s post-tests, was used to compare the statistical differences among multiple groups. *p<0.05, **p<0.01, ***p<0.001

Activation of p38 MAPK pathway plays an important role in the pathogenesis of tissues fibrosis. As mentioned above, the induction of Nr4a1 expression was accompanied with the activation of p38 MAPK phosphorylation in both in vivo and in vitro models of renal fibrosis. As such, we investigated whether Nr4a1 exerted a pro-fibrotic effect through the activation of p38 MAPK. We showed that following TGF-β1 treatment, HK-2 cells transfected with *Nr4a1* siRNA had a significantly lower level of phosphorylated p38 MAPK compared to the cells transfected with Negative Control siRNA (NC siRNA) (Fig. 5A-B). Csn-B treatment alone promoted p38 MAPK phosphorylation (Fig. 5C-D). Notably, treatment with p38 MAPK inhibitor SB203580 dramatically suppressed Csn-B-induced expression of fibrotic protein Fn and Col-I (Fig. 5E-F). Taken together, these findings indicate that Nr4a1 promotes renal fibrosis at least partially through activating p38 MAPK.

**Fig. 6:**
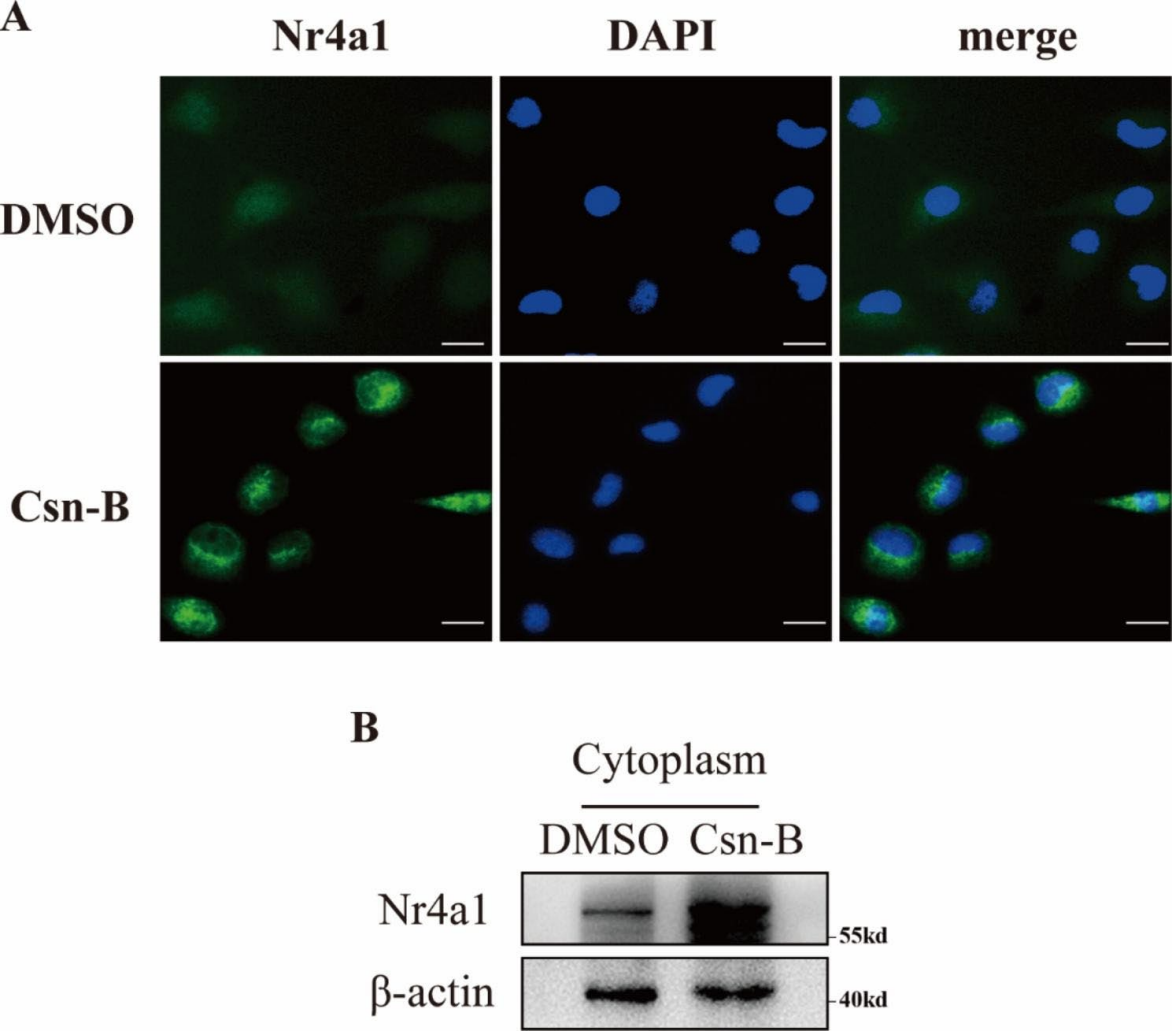
**Csn-B promotes cytoplasmic accumulation of Nr4a1 in HK-2 cells** (**A**) Immunofluorescence analysis of the subcellular expression of Nr4a1 in HK-2 cells following Csn-B treatment (200x, Bar:10 μm). DAPI (blue) indicated the nucleus. (**B**) Western blot analysis of Nr4a1 in the cytoplasmic fraction of HK-2 cells treated with or without Csn-B.

It has reported that Csn-B treatment induced Nr4a1 nucleocytoplasmic translocation(Zhan et al. 2008). We thus assessed the subcellular localization of Nr4a1 in HK-2 cells with or without 10ug/ml Csn-B treatment. Immunofluorescence analysis showed that HK2 cells treated with Csn-B showed a higher level of Nr4a1 and cytoplasmic accumulation of Nr4a1 compared to the cells without Csn-B treatment (Fig. 6A), and western blot analysis verified these findings (Fig. 6B).These results suggests that Csn-B promotes the expression and cytoplasmic accumulation of Nr4a1.

## Discussion

Renal interstitial fibrosis (RIF) is a common pathway to end-stage renal disease regardless of the initial etiology (Ruiz-Ortega et al. 2020), but the molecular mechanism for renal fibrosis remains largely unclear. In the present study, we investigated the role and potential action mechanism of Nr4a1 in renal fibrosis by using kidney biopsies from UUO mouse model of renal fibrosis, and TGF-β1-treated renal tubular cells. The major findings in this study include: First, we found that Nr4a1 was upregulated in renal tubular cells of UUO mice as well as in TGF-β1-treated renal tubular cells, and the levels of Nr4a1 was positively correlated with the degree of interstitial kidney injury. Second, activation of Nr4a1 with a specific agonist Csn-B exacerbated renal fibrosis in UUO mice, and promoted fibrotic changes in TGF-β1-treated renal tubular cells. In addition, we provided further evidence that Nr4a1 promoted renal fibrosis potentially through activating p38 MAPK kinase. Collectively, these findings suggest that activation of Nr4a1-p38 MAPK promotes renal fibrosis.

Nr4a1, a member of the NR4A subfamily of nuclear receptors, is a ligand-activated transcription factor that is highly homologous in gene structure to other NR4A receptors(Saucedo-Cardenas et al. 1997). Similar to other transcription factors, NR4A receptors can bind to target gene promoter regions in the form of monomers, homodimers or heterodimers to regulate gene expression(Wilson et al. 1991; Maira et al. 1999; Philips et al. 1997). In addition, it has been reported that cytoplasmic Nr4a1 can function through protein-protein binding in a transcription-independent manner(Lin et al. 2004). Several recent studies have indicated that Nr4a1 played a role in the progression of fibrotic diseases(Zhou et al. 2018). However, the precise role of Nr4a1 in renal fibrosis remains largely unclear. In the present study, we provided several lines of evidence that activation of Nr4a1 in renal tubular epithelial cells promoted renal interstitial fibrosis. First, upregulation of renal tubular Nr4a1 in UUO mice was associated with the degree of renal fibrosis (Fig. 1B-G). Second, suppress of Nr4a1 expression attenuated TGF-β1-induced expression of fibrotic proteins in renal tubular cells (Fig. 4F-G). Third, activation of Nr4a1 with the specific agonist Csn-B exacerbated renal fibrosis in UUO mice, and promoted TGF-β1-induced fibrotic changes in renal tubular cells (Figs. 2A-E and 4H-I). In consistency with our findings, Nr4a1 deletion was shown to attenuate high glucose-induced renal fibrotic lesions and collagen expression in diabetic nephropathy(Sheng et al. 2018; Zhou et al. 2018). Genetic ablation of Nr4a1 ameliorated alcohol-induced hepatocyte vacuolization, fibrosis and steatosis in mice(Zhou et al. 2018). These findings suggest a profibrotic role of Nr4a in these disease conditions. In a contrast, some studies reported that Nr4a1 is anti-fibrotic(Palumbo-Zerr et al. 2015; Pulakazhi Venu et al. 2021). For instance, Palumbo-Zerr et al. (2015). Another study demonstrated that NR4A1 attenuated fibrotic processes in intestinal myofibroblasts(Pulakazhi Venu et al. 2021). The finding from others and us indicate that NR4A1 may have different or even opposite role in the regulation of fibrosis according to the cell-types and diseased conditions.

Csn-B is a specific agonist of NR4A1 that has been shown to activate NR4A1(Zhan et al. 2008). In the present study, we showed that administration of Csn-B (Selleck) by oral gavage aggravated UUO-induced renal fibrosis. In addition, 50 mg/kg of Csn-B given alone also increase the expression of fibrotic proteins.(Fig. 3D-E). Consistently, Csn-B treatment enhanced TGF-β1-induced fibrotic changes in cultured renal tubular cells, and which was abrogated via suppressing NR4A1 expression (Fig. 3F-I). These in vivo and in vitro findings indicate a profibrotic role of Csn-B in the kidneys. In a sharp contrast, Palumbo-Zerr et al. (2015).The opposite results may be the role of Csn-B that dependable on the cell types and/or disease conditions. Palumbo-Zerr et al. also showed that 13 mg/kg Csn-B (Sigma) treatment also ameliorated UUO-induced renal fibrosis in mice(Palumbo-Zerr et al. 2015). The precise reason for the discrepancy on the effect of Csn-B treatment on UUO-induced renal fibrosis remains unclear, but different dosage of Csn-B treatment may be a potential explanation. In our present study, 50 mg/kg of Csn-B was used in mice, while a much lower dosage (13 mg/kg) was used in the study by Zhan et al. (2008). The different dosage may cause different subcellular expression of Nr4a1, and thus result in different even opposite cellular effect. For instance, Palumbo-Zerr et al. (2015). Emerging evidence also suggested that translocation of Nr4a1 into the mitochondria of cancer cells induces their apoptosis, whereas nuclear Nr4a1 in these same cells may actually promote survival(Lin et al. 2004; Zhou et al. 2014). Another study showed that blockade of the translocation of Nr4a1 to the cytoplasm contributed to bellidifolin-mediated anti-fibrotic effect on isoprenaline-induced myocardial fibrosis(Yang et al. 2021). In the present study, we showed that Nr4a1 mainly localized in the nucleus of renal tubular cells under physiological conditions, and accumulated in the cytoplasm following Csn-B treatment at the profibrotic concentration(Fig. 6A-B), suggesting a potential contribution of cytoplasmic Nr4a1 in facilitated renal fibrosis. However, the potential underlying mechanisms remains to be investigated. Collective, these findings suggest the effect of Csn-B may be determined by its dosage, targeted cell types, and disease conditions. Optimizing the dosage of Csn-B, and specific delivery of Csn-B to target cells are essential for yielding beneficial effect. In addition, the specific role of Nr4a1 in different subcellular sites awaits deeper investigation.

Activation of p38 MAPK has been implicated in the pathogenies of tissue fibrosis (Rhyu et al. 2005; Hung et al. 2016). Some studies indicated that Nr4a1 interacted with p38 MAPK(Hedrick and Safe 2017; Zhou et al. 2014). For instance, Jian et al. showed that LPS induced the exit of nuclear Nr4a1 in lung cancer cells A549, and the cytoplasmic Nr4a1 interacted with p38 MAPK(Jiang et al. 2016). Nr4a1 was also found to interact with p38 in a sepsis model of LPS-induced mononuclear macrophages(Li et al. 2015). In addition, Nr4a1 was involved in DNK-PKcs elevation and p53 phosphorylation in non-alcoholic fatty liver disease (NAFLD) (Zhou et al. 2018). Despite these findings, the potential biological interaction of these two proteins in renal fibrosis remains unclear. In this study, we showed that in both in vivo and in vitro models of renal fibrosis, Nr4a1 induction was accompanied with an increase of p38 MAPK phosphorylation, and moreover, treatment of p38 MAPK inhibitor abrogated Csn-B-induced expression of fibrotic proteins(Figs. 3D and 5E-F), suggesting that Nr4a1-mediated pro-fibrotic effect is at least potentially through activating p38 MAPK. Currently, there is no evidence suggests that Nr4a1 has phosphokinase activity. The precise mechanism underlying the role of Nr4a1 in the regulation of p38 MAPK phosphorylation remains to be investigated.

The next question is with Csn-B-treated mice alone, renal pathological changes were not evident despite there were significantly elevated molecular-levels of fibrotic protein expression as well as p38 phosphorylation levels. We speculated that this probably due to the dosage and or the treatment time. Reference to current models of drug nephrotoxic kidney injury in cisplatin or aristolochic acid-induced renal fibrosis, we found that variable doses of drugs or modeling time had a large impact on the success of model construction and were prone to heterogeneous in renal pathology. Thus, the dosage and the modeling time needs to be further investigated in the future. Furthermore, a limitation of the study is whether this toxicity of dose of Csn-B in the kidney is due to a specific effect of activating Nr4a1 or an off-target effect. It will be interesting to determine the effect of this dosage of Csn-B on kidney in renal tubular cells Nr4a1 knockout mic in future studies.

In the present study, we showed that Csn-B treatment alone induced the expression of fibrotic proteins and p38 MAPK phosphorylation, but did not cause apparent renal pathologies. One potential explanation is the dosage and /or duration of Csn-B treatment is not adequate to induce renal fibrosis. In support of this notion, emerging evidence indicated that the doses and treatment duration of cisplatin or aristolochic acid was critical for triggering renal fibrosis in animal models (Urate et al. 2021; Torres et al. 2016; Katagiri et al. 2016). Thus, it will be interesting to determine whether Csn-B treatment is sufficient to cause renal fibrosis through increasing the dosage and/or during of treatment in future studies.

## Conclusions

In summary, this study provided both in vivo and in vitro evidence supporting that induction of Nr4a1 promotes renal fibrosis potentially through the activation of p38 MAPK pathway. Csn-B treatment aggravated renal fibrosis. As such, Nr4a1 represents a novel target for the prevention and treatment of renal interstitial fibrosis.

## Electronic supplementary material

Below is the link to the electronic supplementary material.

Supplementary Material 1

## Data Availability

The authors agree the data supporting the findings of this study are available upon requests.

## Abbreviations

CGN: Chronic glomerulonephrits
CKD: Chronic kidney disease
Col-I: Collagen-I
Csn-B: Cytosporone B
EMT: Epithelial-to-mesenchymal transition
Fn: Fibronectin
HE: Hematoxylin-eosin
HK-2: Human renal proximal tubular epithelial cells
IHC: Immunohistochemical
IRI: Renal ischemia-reperfusion injury
Nr4a1: Nuclear receptor subfamily 4 group A member
PAS: Periodic acid-Schiff
RIF: Renal interstitial fibrosis
TGF-β1: Transforming growth factor-β1
UUO: Unilateral ureteral obstruction
