# Supplementary material for: Nr4a1 promotes renal interstitial fibrosis by regulating the p38 MAPK phosphorylation
Source: Mol Med. 2023 May 9;29:63. doi: 10.1186/s10020-023-00657-y (PMC10169452; doi:10.1186/s10020-023-00657-y)
Supplement: Supplementary file 1 — Supplementary Material 1 [file 10020_2023_657_MOESM1_ESM.docx]

­**Supplement**


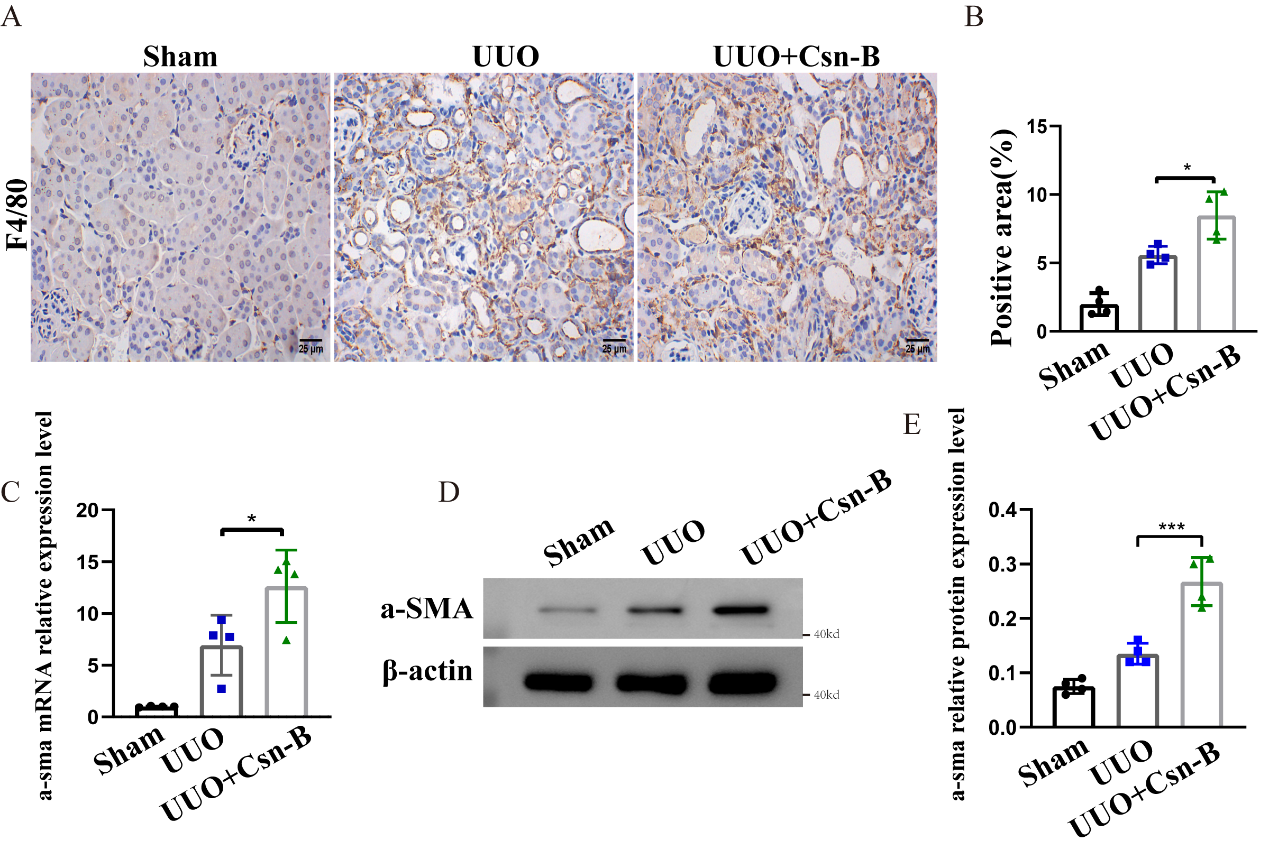


**Supplementary Fig1. Csn-B induces renal infiltration of macrophage and renal expression of α-SMA in UUO mice**

(A) Immunohistochemical analysis of renal infiltration of macrophage F4/80 in kidney tissues (400x magnification, scale bar:25μM) and (B) Quantitative analysis of renal infiltration of macrophage. (C)RT-PCR、(D)Western blot analysis and (E) quantitative analysis of a-sma expression in kidney tissue. One-way analysis of variance (ANOVA), followed by Tukey’s post-tests, was used to compare the statistical differences among Sham、UUO and UUO+Csn-B groups. *p＜0.05，**p＜0.01，***p＜0.001


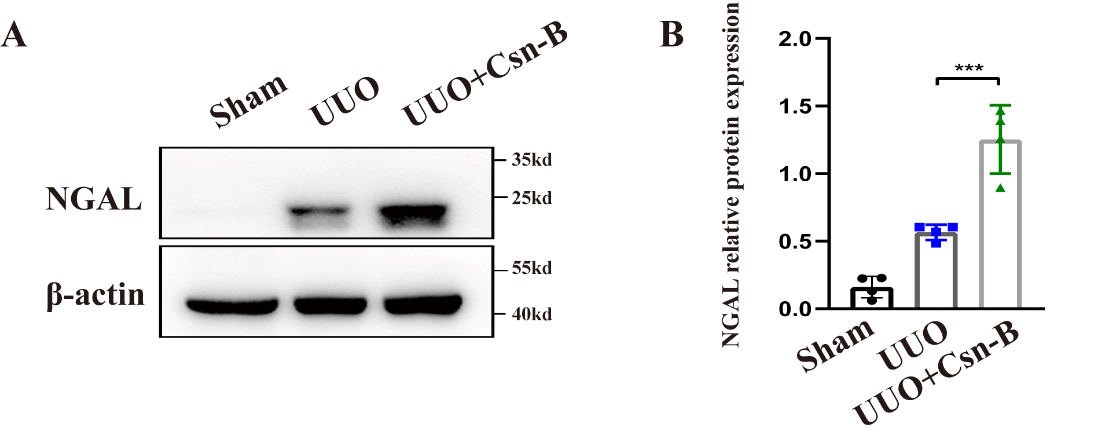


**Supplementary Fig2. Csn-B** **increases the expression of NGAL in UUO mice**

(A)Western blot analysis and (B) quantitative analysis of NGAL expression in kidney tissue. One-way analysis of variance (ANOVA), followed by Tukey’s post-tests, was used to compare the statistical differences among sham、UUO and UUO+Csn-B groups. *p＜0.05，**p＜0.01，***p＜0.001

**
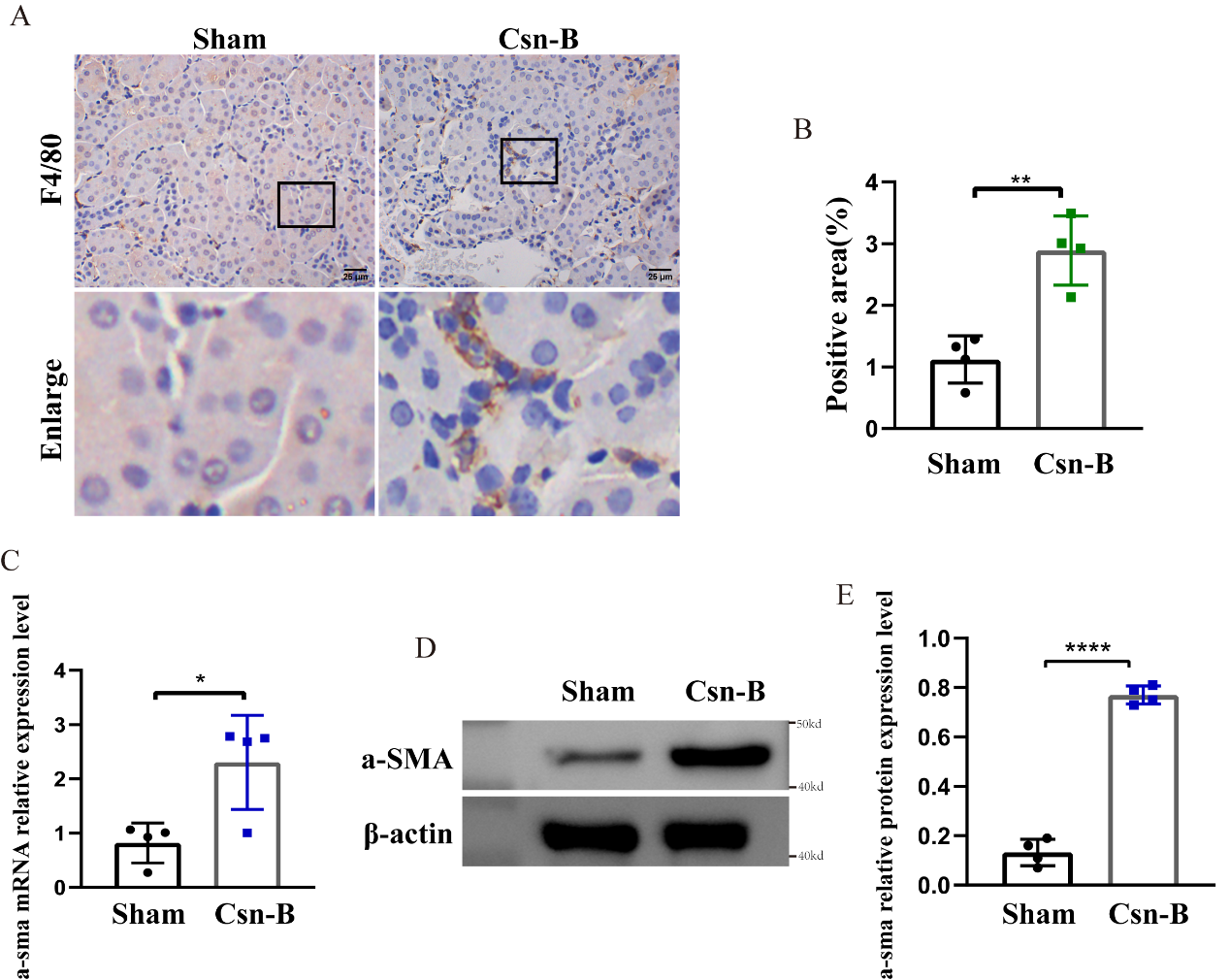
**

**Supplementary Fig3. Csn-B induces renal infiltration of macrophage and renal expression of α-SMA in normal control mice.**

(A) Immunohistochemical analysis of renal infiltration of macrophage F4/80 in kidney tissues (400x magnification, scale bar:25μM). (B) Quantitative analysis of renal infiltration of macrophage. (C) RT-PCR、(D)Western blot analysis and (E) quantitative analysis of a-sma expression in kidney tissue. Unpaired t test was used to compare the statistical differences among sham and Csn-B groups. *p＜0.05，**p＜0.01，***p＜0.001

**
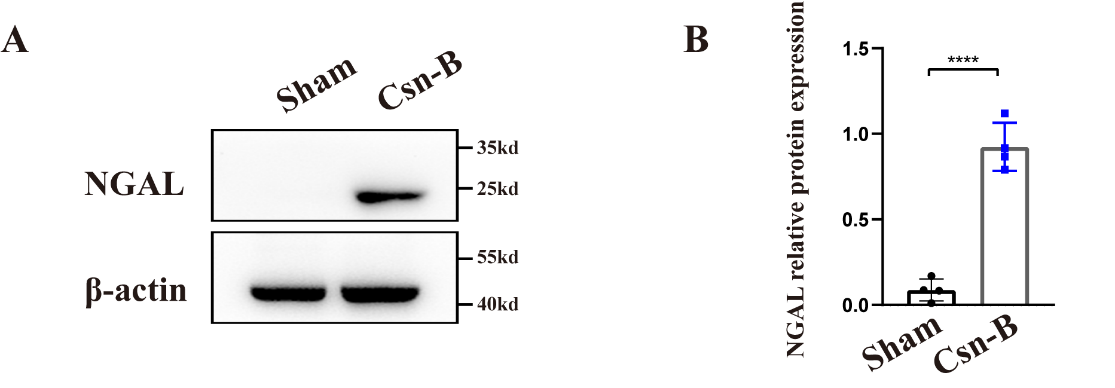
**

**Supplementary Fig4. Csn-B induced renal expression of NGAL in normal control mice**

(A)Western blot and (B) corresponding quantitative analysis of renal expression of NGAL in mice with or without Csn-B treatment. Unpaired t test was used to compare the statistical differences between sham and Csn-B groups. *p＜0.05，**p＜0.01，***p＜0.001

**
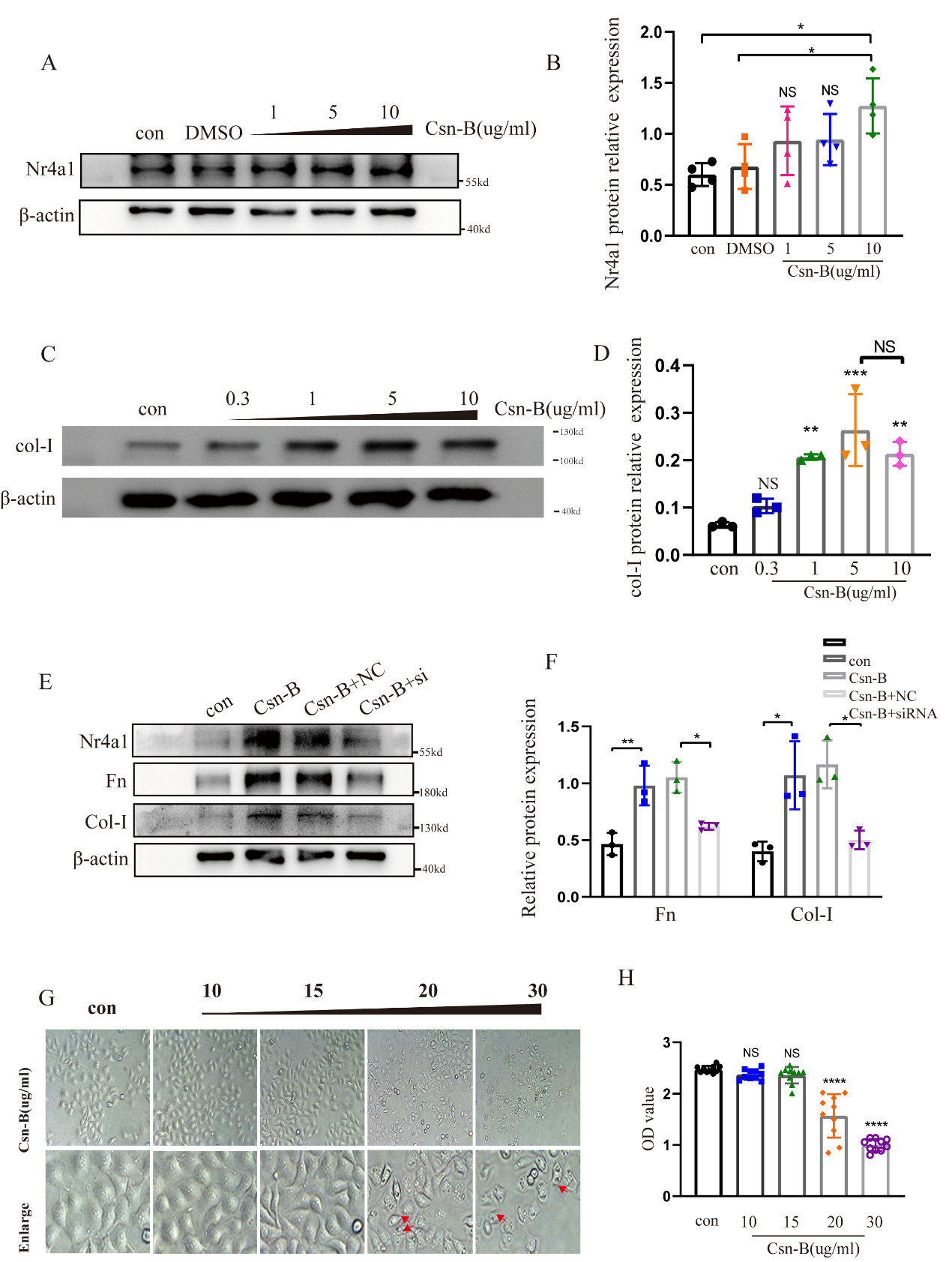
**

**Supplementary Fig5. The effect of different concentrations of Csn-B on HK-2 cells**

(A)Western blot and (B)corresponding quantitative analysis of Nr4a1 expression in HK-2 cells treated with different concentrations(1ug/ml, 5ug/ml, 10ug/ml) of Csn-B for 24 hours. (C)Western blot and (D)corresponding quantitative analysis of col-I expression in HK-2 cells treated with different concentrations (0.3ug/ml, 1ug/ml, 5ug/ml, 10ug/ml) of Csn-B for 24 hours. (E) Western blot and (F)corresponding quantitative analysis of Fn, Col-I expression in HK-2 cells with/without Nr4a1 siRNA expression and 10ug/ml Csn-B treatment. (G) Microscopy analysis of the morphology of HK-2 cells treated with different concentrations (10ug/ml, 15ug/ml, 20ug/ml, 30ug/ml) of Csn-B. (H) CCK-8 analysis of cell activity of HK-2 cell treated with different concentrations of Csn-B (10ug/ml, 15ug/ml, 20ug/ml, 30ug/ml). One-way analysis of variance (ANOVA), followed by Tukey’s post-tests, was used to compare the statistical differences among multiple groups. *p＜0.05，**p＜0.01，***p＜0.001
